# Social uncertainty promotes signal complexity during approaches in wild chimpanzees (*Pan troglodytes verus*) and mangabeys (*Cercocebus atys atys*)

**DOI:** 10.1098/rsos.231073

**Published:** 2023-11-29

**Authors:** Mathilde Grampp, Liran Samuni, Cédric Girard-Buttoz, Julián León, Klaus Zuberbühler, Patrick Tkaczynski, Roman M. Wittig, Catherine Crockford

**Affiliations:** ^1^ The Ape Social Mind Laboratory, Institut des Sciences Cognitives, CNRS UMR 5229, Bron, France; ^2^ Department of Human Behaviour, Ecology and Culture, Max Planck Institute for Evolutionary Anthropology, Leipzig, Germany; ^3^ Taï Chimpanzee Project, Centre Suisse de Recherches Scientifiques, Abidjan, Côte d'Ivoire; ^4^ Taï Monkey Project, Centre Suisse de Recherches Scientifiques, Abidjan, Côte d'Ivoire; ^5^ Department of Human Evolutionary Biology, Harvard University, Cambridge, MA, USA; ^6^ School of Psychology and Neuroscience, University of St Andrews, St Andrews, UK; ^7^ Cooperative Evolution Laboratory, German Primate Center, Göttingen, Germany; ^8^ Institute of Biology, University of Neuchâtel, Neuchatel, Switzerland; ^9^ School of Biological & Environmental Sciences, Liverpool John Moores University, Liverpool, UK

**Keywords:** social complexity, multisensory signalling, fission–fusion dynamics, signal combination, behavioural flexibility

## Abstract

The social complexity hypothesis for the evolution of communication posits that complex social environments require greater communication complexity for individuals to effectively manage their relationships. We examined how different socially uncertain contexts, reflecting an increased level of social complexity, relate to variation in signalling within and between two species, which display varying levels of fission–fusion dynamics (sympatric-living chimpanzees and sooty mangabeys, Taï National Park, Ivory Coast). Combined signalling may improve message efficacy, notably when involving different perception channels, thus may increase in moments of high social uncertainty. We examined the probability of individuals to emit no signal, single or multisensory or combined (complex) signals, during social approaches which resulted in non-agonistic outcomes. In both species, individuals were more likely to use more combined and multisensory signals in post-conflict approaches with an opponent than in other contexts. The clearest impact of social uncertainty on signalling complexity was observed during chimpanzee fusions, where the likelihood of using complex signals tripled relative to other contexts. Overall, chimpanzees used more multisensory signals than mangabeys. Social uncertainty may shape detected species differences in variation in signalling complexity, thereby supporting the hypothesis that social complexity, particularly associated with high fission–fusion dynamics, promotes signalling complexity.

## 1. Introduction

The social complexity hypothesis for the evolution of communication posits that social and communicative complexity may be intimately related, as communication may allow individuals to flexibly respond to social challenges and uncertainty in complex social environments [1–5]. Social or communicative complexity may arise from an increase in the number and types of its components and their interactions [2,6], where heterogeneous and unpredictable systems may be more complex than homogeneous and ordered ones [7]. It has been proposed that societies with high variability across different types of relationships probably increase levels of social complexity experienced by individuals [8]. This parameter may be estimated by an increase in group size [9–11], or more precisely by an increase of the level of differentiation in social relationships [5,6,10,12–16], or the number of social roles [17]. Further, high variation within relationships, namely changes in the patterning of social interactions experienced by individuals over time, may also increase social complexity [8]. For instance, high variation in the social environment, such as caused by high entropy in the audience composition, may have implications for the frequency of interactions between group members and the role of third-parties [3,18–22]. However, defining, quantifying and comparing degrees of social and communicative complexity, particularly across species, is challenging and remains an ongoing debate [1,6,12,23–25], notably depending on the focus and the scale of complexity measurements [26]. For instance, among macaque species, less predictable and egalitarian social structures may be associated with higher levels of vocal and facial signal variability [24,27]. Across primate species, more despotic dominance hierarchies may relate to larger hierarchy-related vocal repertoire sizes [23], or high vocal variability in agonistic contexts particularly in large multimale, multifemale groups [28]. On a dyadic scale, uncertainty in whether the outcome of a social approach towards another conspecific will result in aggression [29], may directly promote the need for communicative complexity [1,5,30–32] as a way to improve message transmission or interpretation [32,34]. In line with this hypothesis, within species and between relationships, it was previously shown that signalling complexity may increase during interactions between individuals who are close in dominance rank or are unfamiliar, as such relationships are associated with uncertain outcomes [23,31,32]. However, studies within and between species assessing how individuals change their signal complexity when faced with different contexts of social uncertainty are lacking, thereby restricting our ability to assess the relative contribution of phylogeny, ecology, and the impact of changes in social complexity during daily life in shaping communicative systems [1,35].

Social contexts such as post-conflict approaches with a former opponent or third parties may generate uncertainty about their outcome, given a risk of renewed or redirected aggression [29,36]. Here, certain behavioural strategies, such as initiating a grooming interaction with, or greeting a former opponent [37,38], may serve as a means to reduce tension, enable self-protection, or repair relationships [36,37,39–44]. Further, a feature of social systems considered to substantially increase social complexity and uncertainty is fission–fusion dynamics [15,45,46]. Fission–fusion dynamics relate to the level of social cohesiveness of a group, and describe variation in the availability of partners [19] and associated social strategies [20,21,45]. The high fluctuation in group cohesion in species with high fission–fusion dynamics also impacts the opportunities for a given individual to witness third-party interactions and gather information about interaction history and individual behavioural strategies [22,46,47]. Because group members do not permanently associate in these species, individuals may need to re-assess social and hierarchical relationships after periods of separation. Thus when parties are merging (fusion), individuals may adjust their behaviour to accommodate an increased risk of social conflict [30,48–50]. Long-distance communication between dispersed group members (namely inter-party communication, where a ‘party’ is a subgroup of community members) may update information about the identity, the location, or the activity of other, out-of-sight, group members [51,52]. Therefore, inter-party communication may be associated with changes in within-party social strategies, for instance instigating the choice of whether to change parties, or promoting other possible shifts in association as one's own or others’ allies or competitors identify themselves nearby [21,38,53].

Social animals flexibly employ various strategies and signals while approaching other members of their group, including ‘greeting’, ‘attention-getter’ and ‘benign intent’ signals [30,54–57]. These signals are known to regulate social relationships [58], from the negotiation of tolerance and conflict prevention to coordination or cooperation [39,55,59,60], particularly in species with high levels of fission–fusion dynamics [30,49,61]. Across birds, arthropods and mammals, combining signals or sensory modalities limits communication ambiguity and failures, notably during disturbing environmental conditions [33,34,62]. In primates, signallers have been reported to emit combined vocal signals in socially uncertain interactions, in relation to variation in dominance rank [30,54] and bondedness [54], and in noisy social environments [63]. Signallers have been reported to combine vocal and visual signals in relation to dominance interactions [30] and combine modalities of signal production in relation to interactions with unfamiliar individuals [32]. Although an increase of signal combination or compositionality may represent an optimized and possibly less complex form of signalling than an increase of signal variability in human language sciences [26], it may also allow an expansion of repertoire size [64–66]. It seems rare across species but may be particularly developed in highly social species [67,68].

One way to pin down the impact of social uncertainty on signalling complexity, is to assess whether shifts in the social uncertainty that individuals face through daily life changes their signalling complexity. Specifically, we tested four contexts of social uncertainty that may particularly stimulate signalling probability and complexity during social approaches [32,34], related to the need to re-assess a relationship and to reduce the risk of aggression and/or losing a social partner. The four contexts included those generated by fission–fusion dynamics, such as (i) fusion and (ii) inter-party communication, as well as contexts of post-conflict with (iii) a former opponent, or (iv) a third-party individual. Studies have identified different aspects of signalling complexity, of which we focus on two: (a) multisensory signals—the use of signals of more than one sensory modality, specifically visual and auditory, and (b) combined signals—combining more than one signal type simultaneously or into a sequence (independent of the sensory modality), as signal complexity may increase with an increasing number of signal components [4].

To evaluate the effects of social uncertainty on signalling complexity independently of environmental variation, we studied sympatric species with different social systems (e.g. degrees of fission–fusion dynamics) exposed to the same low visibility, noisy, forest habitat: sooty mangabeys (*Cercocebus atys atys*) and western chimpanzees (*Pan troglodytes verus*) from the Taï forest, Ivory Coast [21,38]. Hence, we could examine the impact of social dynamics while keeping habitat effects on signalling strategies constant. To further limit the potential impact of the physical environment on signalling complexity, we only examined signal production in close-range dyadic communication (social approaches within 2 m, a distance allowing for full visual and auditory contact between partners).

Both species exhibit certain common social features; i.e. large multimale, multifemale groups, primarily maternal care, a polygamous mating system, differentiated social relationships, and a moderately steep dominance hierarchy (electronic supplementary material, table S1 and figure S1). Both populations are also largely terrestrial and consume ephemeral fruits, thus, both experience competition for resources that may select for social and communication complexity [28]. A previous study found that captive mangabeys increase the use of signal combinations predominantly in the visual and auditory channels during aggression and playful contexts [69], although their population displayed a sex ratio biased towards males non-representative of wild populations. In wild chimpanzees, combinations of visual and auditory signals were mostly associated with affiliative and aggressive social interactions [70]. Most notably, during aggressive fusion events, chimpanzees increase the number of signal types, and sensory modalities during greeting signals [30]. Thus, both species may use complex, combined and multisensory, signalling during socially uncertain contexts.

Wild chimpanzees show higher fission–fusion dynamics; i.e. higher frequency, stability and cohesiveness of temporary parties [18,71], compared with mangabeys [21], which probably generate differences in social complexity [19,45]. Further, previous studies demonstrated that social relationships are more consistent over time in mangabeys compared with chimpanzees, potentially in relation to their matrilineal social structure generating a strong reliance on kin bonds [72]. Considering these species differences in social organization and structure, we examined the impact of socially uncertain contexts within species, and then compared standardized effect sizes between species. In summary, we predicted that within species, social approaches during social contexts with high social uncertainty compared to other contexts, would elicit greater signalling probability, specifically regarding combined signal types and multisensory signals, in line with the hypotheses that such signals may disambiguate messages and/or improve message integration [32,34,73]. We also considered variation in the production of single signalling, as single signals may also reduce message ambiguity in socially uncertain contexts [39]. Further, as high fission–fusion is associated with increased social uncertainty [46], between-species we expected overall greater signalling complexity in chimpanzees than mangabeys [45,74].

## 2. Methods

### 2.1. Study site and subjects

M.G. conducted this study in the Taï National Park (5°52′ N, 7°20′ E, Ivory Coast) [75]. M.G. carried out focal observations [76] on two individuals per day for 6 h each, from dawn to midday and then from midday to dusk. Focal subjects were all male and female adult and sub-adult individuals in two wild groups per species of mangabeys and chimpanzees, all habituated to human presence (chimpanzees greater than 9 years = 38 individuals, and mangabeys greater than 2 years = 51 individuals; further details in the electronic supplementary material, S1 and table S2). While three groups were at the Taï Chimpanzee Project (TCP), the second group of mangabeys was at the Taï Monkey Project (TMP), which ranges approximately 4 km away from the TCP group of mangabeys [75,77]. The order of focal follows was chosen pseudo-randomly (chimpanzees: mean ± s.d. = 41 ± 9 h individual^−1^, mangabeys: 22 ± 10 h individual^−1^), with a priority given to less observed individuals in order to balance observation hours across individuals.

### 2.2. Data collection

#### 2.2.1. Signalling behaviour

During a focal follow, when non-focal individuals approached (entered a 2 m perimeter of) the focal individual, or *vice versa*, M.G. collected data on the identity of these individuals and the signals directed to/from the focal individual, within a maximum of 10 s after the approach in cases when there was no change of receiver behaviour. We define ‘signals’ as gestures and vocalizations that have been previously described in each species, that are considered to transmit information to others, whether intentionally or unintentionally [69,70,78,79]. Exceptions were ‘peer’ and ‘withdraw’ that could be rather social cues than signals (i.e. informative traits but that have not been under selection for facilitating transmission of this information, as defined in [80]), as distinguishing social cues from signals may not always be straightforward [32]. ‘Combined signals’ were defined as visual and/or auditory signals that were produced continuously (less than 1 s gap between them, either more than one auditory and/or visual signal) during the time of the approach. We only considered signals from the individual who initiated signal production. M.G. noted each signal type emitted but not repetitions of the same signal type. For combined signals, we did not consider the order of emission, simply whether one or more than one signal type (e.g. bark, grunt, ground slap) was emitted, and the sensory modality of the signal type (visual, auditory, or multisensory). Data during approaches and throughout focal follows were collected using a smartphone device (Caterpillar) and the Cybertracker software (https://cybertracker.org/).

In order to account for the possibility that the observer increased the likelihood to detect signals during approaches over time owing to her experience we used date as a control factor in our models. *Visual signals* included in chimpanzees: arm raise; and in both species: head movements, standstill displays, withdrawing, bowing/crouching, peering, present body and genitals, extend limb and throw arm [69,81,82]. *Auditory signals* included vocalizations (chimpanzees: bark, hoo, pant-grunts, pants, pant-hoots, whimper, pant-screams or barks [30,79]; mangabeys: twitter, growl, copulation call [78]; both species: grunt and scream) and non-vocal sounds (chimpanzees: lip-smacking, raspberry blowing, teeth-clacking) [83]. *Multisensory signals* included single type signals, namely body and arm gestures using body parts, objects, or ground to make sound (e.g. shaking a branch) and combinations of both auditory and visual modalities in combined signals (see the electronic supplementary material, table S3 and S2.1 for further details about signal categories).

We conducted several reliability tests using Cohen's Kappa method [84]. We compared the reliability of coding the signalling variables using videos of approaches, between M.G. and other researchers experienced with signalling in mangabeys (test 1) and chimpanzees (test 2). Because, only 2% of approaches could be filmed, we estimated the reliability of real-time coding and video-coding by M.G. using approaches concurrently collected on video and the Cybertracker software, in mangabeys (test 3) and in chimpanzees (test 4). As the chimpanzee vocal repertoire is graded [79], we compared the reliability of coding a vocal sequence as ‘ single signal’ versus ‘combined signals’ with a listening-only method by M.G. and a combined approach of listening and inspecting call spectrograms by another researcher (test 5). Given a low percentage of approaches that could be reliably recorded with both a camera and the Cybertracker, we used additional videos collected by other researchers, and estimated the reliability of coding signalling variables during approaches by M.G. after watching a video once (to simulate live-coding), and then re-watching it several times (as is possible with video data), in mangabeys (test 6) and in chimpanzees (test 7).

Overall, these tests indicated good accuracy of coding the signalling variables across researchers and methods (test 1: к = 0.80 and 0.88; test 2: к = 0.88 and 0.76; test 3: к = 0.93 and 0.97; test 4: к = 0.84 and 0.87; test 5: к = 0.74; test 6: к = 0.90 and 1; test 7: к = 0.95 and 0.93). Further, there was a representative overview of the diversity of signals across the recordings (test 1: *n*_No_ = 44 without signalling, *n*_single_ = 31 with a single signal, *n*_combined_ = 18 with combined signals; test 2: *n*_No_ = 31, *n*_single_ = 19, *n*_combined_ = 25; test 3: *n*_No_ = 28, *n*_single_ = 22, *n*_combined_ = 13; test 4: *n*_No_ = 10, *n*_single_ = 7, *n*_combined_ = 24; test 5: *n*_single_ = 38, *n*_combined_ = 55; test 6: *n*_No_ = 17, *n*_single_ = 26, *n*_combined_ = 21; test 7: *n*_No_ = 17, *n*_single_ = 21, *n*_combined_ = 26; see the electronic supplementary material, S2.2 and tables S4–S7 for further details about reliability tests).

As we wanted to focus on a form of conflict management that will reduce social uncertainty, the social threat and the risk of conflict or conflict renewal, we excluded approaches when the signaller showed agonistic behaviour (i.e. chased, charged, hold, bite, hit, jumped on or pulled the receiver). However, because some behaviours are ambiguous (i.e. poke, tap, push, grab a body part, bipedal swagger or jump towards), we also removed approaches when the partner fled as a response to the signaller's behaviour.

#### 2.2.2. Social contexts

We considered four contexts of social uncertainty: (i) fusion, (ii) inter-party communication, (iii) post-conflict context with an opponent, and (iv) post-conflict context with a third-party individual (see the electronic supplementary material, S2.4 and table S8 for more details about social contexts):

(i) *fusion*: a new party was assigned when at least one individual or a sub-group split from or joined others (‘party’ defined as individuals within visibility of each other, 30 m on average [21]). We defined approaches during fusions as those occurring between two individuals during a fusion event, i.e. as the two parties were in the process of merging, until individuals resumed their activity. Approaching individuals were in different parties prior to the fusion event. If more than one approach occurred per dyad in this context, only the first approach was categorized as ‘fusion’ (chimpanzees: *n* = 430, mangabeys: *n* = 20; further details in the electronic supplementary material, section S2.4);
(ii) *inter-party communication*: both species may form sub-groups, eliciting inter-party communication, such as, ‘pant-hoots’ and ‘drums’ in chimpanzees [52,79,85], and ‘twitters’ and ‘whoop gobbles’ in mangabeys [78]. We assigned the context ‘inter-party communication’ to approaches between same-party members that occurred within 1 min after inter-party communication events. These included hearing long-distance calls or buttress drums from other parties of the same community in chimpanzees, and ‘whoop gobbles’ in mangabeys, and all long-distance calls within the party in reply to another party or that elicited a reply by another party, chimpanzees: *n* = 95/2589, mangabeys: *n* = 26/402);
(iii) *post-conflict context with an opponent*: the first approaches between the focal individual and its former agonistic opponent (chimpanzees: *n* = 78, mangabeys: *n* = 434) were detected for this context. We only used observations with continuous focal visibility between the conflict and the approach event (average ± s.d.: 14 ± 24 min); and
(iv) *third-party post-conflict context*: we also detected the first approach, initiated, or received by the focal individual after experiencing a conflict, towards a third-party individual who was not involved in the previous conflict (chimpanzees: *n* = 127, mangabeys: *n* = 667, average ± s.d.: 3 ± 3 min). The maximum duration for post-conflict approaches with a third party was 2 h 14 min, therefore, we decided to only consider post-conflict approaches with a third-party individual when the duration between the conflict and the approach was below 10 min, as post-conflict mechanisms mainly occur during this window of time [86], thus avoiding that other social events may have triggered observed behaviours (75% of approaches occurred within 10 min in our data for both species; see the electronic supplementary material, figure S3).

### 2.3. Estimation of social relationship strength and dominance hierarchy

We additionally controlled for the potential impact of social and dominance relationships on signal complexity, which have provided contrasting results in previous studies. Indeed, social relationship strength may decrease social uncertainty between conspecifics, but also be associated with increased cooperation, which may have opposite effects on signal complexity [49,54,55,63,87]. In terms of dominance rank, social uncertainty, and thus signal probability or complexity during approaches, may increase either between closely ranking individuals [31] or with rank difference as a way to acknowledge another's dominance or communicate benign intent, when emitted by lower or higher-ranking individuals, respectively [23,30,54,55].

We used a grooming index (GI), based on the composite sociality index [88], using the focal rates of grooming initiation and duration per field season, to account for variation in social relationship quality across dyads (more ‘affiliated’ partners showing higher GI values). The dominance hierarchy of each group was estimated by using long-term data on supplants (mangabeys) and pant-grunts (chimpanzees: greeting calls directed up the hierarchy [89]) for all four groups and applying a modification of the package Elo-rating [90] (developed by Foerster *et al*. [91]; see [21] and the electronic supplementary material S3, table S9 and figure S4 for details).

### 2.4. Statistical analyses

#### 2.4.1. General procedure

Statistical analyses and data preparation were conducted in R 4.1.2 [92] using the RStudio Interface [93]. We used multinomial mixed-effect models (categorical family and logit link function; see the electronic supplementary material, S4 for details) with Bayesian estimation using the ‘brms’ package [94].

We included the random effects for ‘signaller’ and ‘receiver’ identities in these models, to account for repeated sampling of the same individuals and imbalanced sampling of individuals in either role, and the ‘dyad’ identity to account for dyad-level factors that may not explicitly be accounted for in the GI or the dominance rank. We also included a random effect intercept for a variable of ‘group/day’ identity. This allowed us to account for group-level factors not explicitly accounted for in our fixed effects, such as food availability, or social tension, that may influence signalling and/or the responses to those signals, but also the potential for the human observer to increase detection skills of ‘combined signals’ over time (see the electronic supplementary material, figure S7 showing posterior distributions of the standard deviations of the random effects, and suggesting no influential effect).

We estimated collinearity using the function *vif* from the ‘car’ package [95]; the maximum variance inflation factor was 1.02, indicating no issues with collinearity. All models converged (Rhat < 1.01) and we checked trace plots and the *pp_check* plots of ‘brms’ to validate all models (electronic supplementary material, figures S5 and S6). Model plots were generated using the package ‘ggplot2’ [96] and the *conditional_effects* function of ‘brms’. When the 95% of the credible interval of an estimate did not overlap 0, we considered that effect was strongly supported by our data, when only the 89% of the credible interval of an estimate did not overlap 0, we considered that effect was weakly supported by our data and showed uncertainty, when the 89% credible interval was overlapping 0, we considered that the effect was not supported by our data. We reported *p+* and *p−* as the percentages of posterior distribution in support for the direction of the estimate.

#### 2.4.2. Models for species comparison of probability of signalling style during approaches

We tested the effect of species (chimpanzee or mangabey) on the likelihood of emitting a signal, and increasing its complexity during each approach event in two models (chimpanzees: *n* = 3699 approaches, mangabeys: *n* = 7054 approaches). In a first model, we examined the probability of emitting single, or combined signals compared to no signal, the response variable was categorized into ‘no signal’, ‘single signal’ or ‘combined signals’. In a second model, we examined the probability of emitting, visual, auditory or multisensory signals compared to no signal, the response variable was categorized into ‘no signal’, ‘visual’, ‘auditory’ or a ‘multisensory’ signal (as either a single or combined signal). We indicated the detected magnitude of change (%) for each signalling style (i.e. single, combined, visual, auditory, multisensory) between species.

#### 2.4.3. Models on the impact of socially uncertain contexts on signalling style during approaches

We fitted two models per species, one for each level of signal complexity (four models, i.e. unisensory versus multisensory signals, single versus combined signals). For each approach event, the response variable had three possible categorical values: no signal, single (whether signal type or sensory modality), or combined (whether signal types or sensory modalities). The reference category used in these models was first ‘no signal’ then was relevelled to ‘unisensory’ or ‘single type’ to get the probability of emitting multisensory versus unisensory signal or combined versus single signal. We tested the predictors of the social context (presence or absence of socially uncertain contexts) on the response variable (the probability of emitting a signal or emitting a complex signal). Contexts were represented in separate predictor variables as binomial variables (presence/absence of each context). While contexts were not mutually exclusive, overlapping contexts represented a small percentage: maximum in chimpanzees: 11.5% of post-conflict events with a former opponent occurred during fusions; maximum in mangabeys: 11.5% of inter-party communication events were also post-conflict approaches with a third party (electronic supplementary material, table S8). We indicated the detected magnitude of change (%) for each signalling style (i.e. single, combined, unisensory, multisensory) between approaches in the presence or absence of a socially uncertain context. Assessing the magnitude of change is relevant for evolutionary theories: if a situation is a particular catalyst for expansion of usage of a certain type of signal (e.g. social uncertainty promotes the use of combined signals), this could promote selection of the usage of such signalling.

We controlled for the relative dominance rank of the signaller as a fixed effect (i.e. categorical variable: signaller is lower-ranking or higher-ranking than the receiver), as independently of the rank distance, lower-ranking signallers may always be facing a higher risk of aggression than higher-ranking ones, and we controlled for the relationship strength between the signaller and receiver by adding the GI as a fixed effect. GI values were *z*-transformed with a mean of zero and a standard deviation of one, in order to more easily interpret their estimates. Chimpanzees and mangabeys differ in their patterns of sex-based dispersal, with male and female philopatry observed respectively in each species [75,77]. Thus, we included the fixed effect of sex combination (sex of the signaller-sex of the receiver, categorical variable with four levels: female-female, female-male, male-female, male-male), as it may influence patterns of greetings during approaches [97]. We also included a fixed effect of group identity to account for group-specific social dynamics unaccounted for in our other fixed effects, such as rank stability or social tolerance levels [98]. Lastly, we could also include the random slopes of the scaled GI for the random effects of the ‘signaller’ and ‘receiver’, the scaled GI and the sex combination for the random effect of ‘group/day’ and the correlations between the random intercepts and slopes.

## 3. Results

### 3.1. Between species comparison of signalling style used during approaches

#### 3.1.1. Probability of emitting single or combined signals

**Figure 1.**
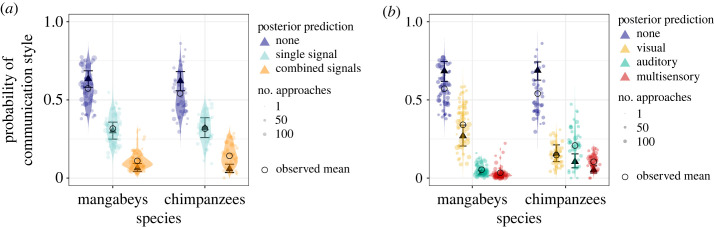
Comparison between chimpanzees and mangabeys in general signalling style during social approaches. (*a*) Probability of emitting single or combined signals. (*b*) Probability of emitting visual, auditory or multisensory signals. Comparison across all (non-agonistic) approaches between the two species. Triangles: posterior means; error bars: 95% credible intervals; dots: individual probabilities from the raw data (size: number of approaches sampled per individual), violin: predicted individual probabilities from the posterior distribution of the model.

Across all approaches, we found no consistent species difference in the probability to emit combined versus single signal types during an approach (single: estimate: 0.08, 95% credible interval (CI) [−0.3, 0.48], *p +* = 66.1%; combined: −0.08, 95% CI [−0.7, 0.54], *p−* = 60.7%; figure 1*a*; detailed results in the electronic supplementary material S5 and table S10).

#### 3.1.2. Probability of emitting visual, auditory or multisensory signals

We found a species difference in the probability to emit more complex signals when considering the production of multisensory signals (figure 1*b*; detailed results in the electronic supplementary material, S5, figures S8–S10). Irrespective of the number of signal types used, chimpanzees were 355% more likely to use auditory signals (estimate: 1.16, 95% CI [0.55, 1.75], *p +* = 100%) and 333% more likely to emit multisensory (both visual and auditory modalities) signals than mangabeys (1.37, 95% CI [0.8, 1.95], *p +* = 100%). Overall, 5.10% of approaches in chimpanzees (*n* = 189) compared with 0.96% in mangabeys (*n* = 68) included multisensory signal types. In chimpanzees, this mainly included using objects to make sound, such as branches, leaves, the ground, or body parts [56]. Mangabeys were 108% more likely to emit visual signals than chimpanzees (108% increase, −0.57, 95% CI [−1.1, −0.05], *p−* = 98.3%). Only visual signals were emitted by mangabeys in 2305 of 2805 approaches with signals and in chimpanzees in 580 of 1780 approaches with signals. Only auditory signals were emitted by mangabeys in 322 of 2805 approaches with signals and by chimpanzees in 793 of 1780 approaches with signals. As approaches with a single multisensory signal type were rarely found in either species (mangabeys: 13 out of 7054, 0.18% of approaches; chimpanzees: 50 out of 3699, 1.35%), we could not include these as a separate level in the analysis.

### 3.2. Impact of socially uncertain contexts on signalling style during approaches

#### 3.2.1. Fusion context

During fusions compared with other contexts, in both species, social approaches were considerably more likely to include signals, with chimpanzees additionally increasing the likelihood to use multisensory and combined signals (electronic supplementary material, tables S11–S14).

**Figure 2.**
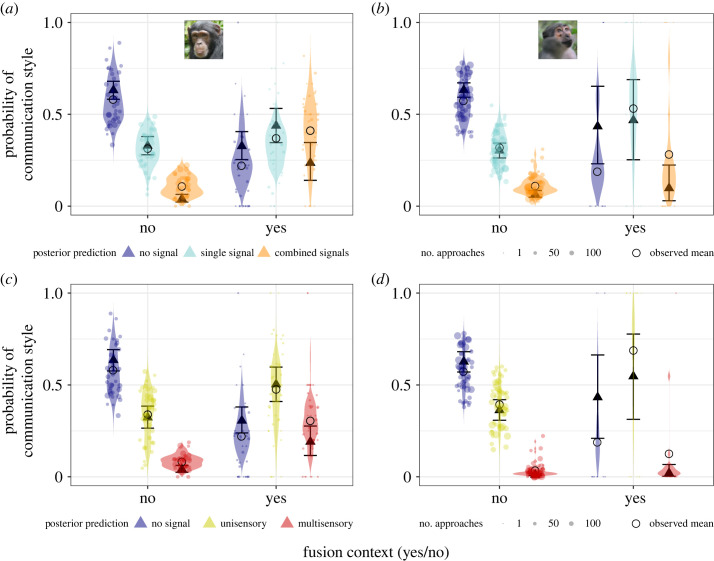
Fusion contexts markedly increased signalling complexity during social approaches in chimpanzees, and increased signalling probability in mangabeys. (*a* and *b*) Probability of emitting single and combined signals during approaches, (*c* and *d*) probability of emitting unisensory (visual or auditory) and multisensory signals during approaches: in chimpanzees (left) and mangabeys (right). Triangles: posterior means; error bars: 95% credible intervals; dots: individual probabilities from the raw data (size: number of approaches sampled per individual per context), violin: predicted individual probabilities from the posterior distribution of the model.

Chimpanzees were 4% more likely to produce single signals (single versus no signal, estimate: 0.93, 95% CI [0.63, 1.23], *p +* = 100%) and 278% more likely to produce combined signals in fusion compared with non-fusion contexts (combined versus no signal, 2.4, 95% CI [2.06, 2.75], *p +* = 100%; combined versus single signal: 1.43, 95% CI [1.13, 1.73], *p +* = 100%; figure 2*a*). Chimpanzees were 39% more likely to emit unisensory signals (unisensory versus no signal, 1.16, 95% CI [0.88, 1.45], *p +* = 100%), and 240% more likely to emit multisensory signals in fusion compared with non-fusion contexts (multisensory versus no signal, 2.21, 95% CI [1.85, 2.57], *p +* = 100%; multisensory versus unisensory signal: 1.02, 95% CI [0.73, 1.32], *p +* = 100%; figure 2*c*).

There was some support that mangabeys were 69% more likely to emit single signals in fusions compared with non-fusion contexts (89% CI not overlapping 0: single versus no signal, 0.87, 95% CI [−0.1, 1.88], *p +* = 96.0%). Mangabeys were 76% more likely to emit unisensory signals in fusions compared with non-fusion contexts (unisensory versus no signal, 0.86, 95% CI [−0.12, 1.86], *p +* = 95.3%). We found no clear evidence that mangabeys were more likely to emit more combined signals in fusion compared with non-fusion contexts (combined versus no signal: 0.78, 95% CI [−0.44, 1.91], *p +* = 90.1%; combined versus single signal: 0.11, 95% CI [−0.98, 1.14], *p +* = 58.1%; and multisensory versus no signal: 0.85, 95% CI [−0.75, 2.32], *p +* = 86.2%; multisensory versus unisensory signal: 0.47, 95% CI [−1.03, 1.86], *p +* = 75.7%; figure 2*b*,*d*).

#### 3.2.2. Inter-party communication context

Inter-party communication events clearly increased signalling probability and complexity in chimpanzees during social approaches, but were more likely to decrease signalling complexity in mangabeys (electronic supplementary material, tables S11–S14).

**Figure 3.**
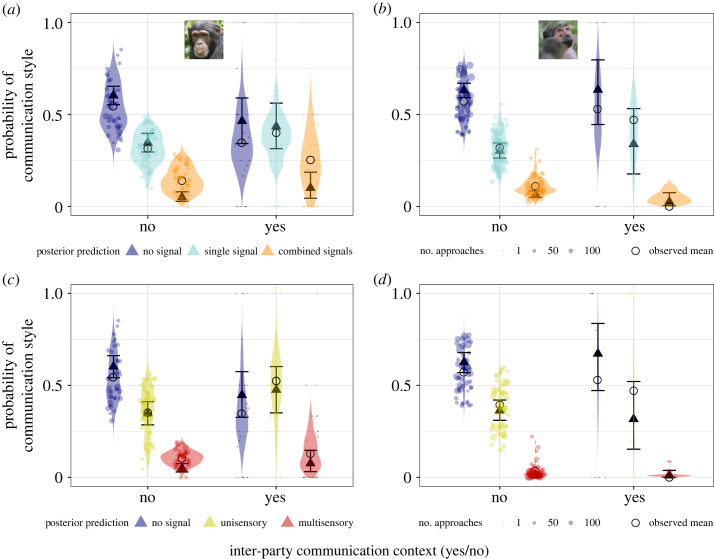
Inter-party communication events increased signalling probability and complexity during social approaches in chimpanzees, but decreased signalling complexity in mangabeys. (*a* and *b*) Probability of emitting single and combined signals during approaches, (*c* and *d*) probability of emitting unisensory (visual or auditory) and multisensory signals during approaches: in chimpanzees (left) and mangabeys (right). Triangles: posterior means; error bars: 95% credible intervals; dots: individual probabilities from the raw data (size: number of approaches sampled per individual per context), violin: predicted individual probabilities from the posterior distribution of the model.

Following inter-party communication events compared with contexts not following inter-party communication events, chimpanzees were 19% more likely to produce single type signals (single versus no signal, 0.51, 95% CI [0, 1.01], *p +* = 97.5%) and 63% more likely to produce combined signals (combined versus no signal, 0.97, 95% CI [0.32, 1.62], *p +* = 99.8%; combined versus single signal: 0.46, 95% CI [−0.13, 1.05], *p +* = 93.6%; figure 3*a*). Further, following inter-party communication events compared with approaches without these events, chimpanzees were 35% more likely to emit unisensory signals (unisensory versus no signal, 0.64, 95% CI [0.15, 1.13], *p* + = 99.6%), and there was some support that chimpanzees were 29% more likely to emit multisensory signals (89% CI not overlapping 0: multisensory versus no signal, 0.7, 95% CI [−0.06, 1.41], *p +* = 96.9%; multisensory versus unisensory signal: 0.12, 95% CI [−0.56, 0.76], *p +* = 64.1%; figure 3*c*).

There was some support that mangabeys were 56% less likely to emit combined signals after inter-party communication events compared with other contexts (89% CI not overlapping 0 for combined versus no signal, −1.17, 95% CI [−2.66, 0.17], *p* *−* = 94.9% and for combined versus single signal: −1.14, 95% CI [−2.67, 0.2], *p* *−* = 94.3%; figure 3*b*), but we found no evidence that these events affected the probability to emit single signals (single versus no signal, 0.09, 95% CI [−0.74, 0.92], *p +* = 58.3%). We also found no clear evidence that these events affected the probability to emit unisensory signals (unisensory versus no signal, −0.22, 95% CI [−1.07, 0.62], *p* *−* = 70.0%) nor the probability to emit multisensory signals (multisensory versus no signal, −0.45, 95% CI [−2.19, 1.18], *p* − = 68.5%; multisensory versus unisensory signal: −0.37, 95% CI [−2.1, 1.28], *p* − = 65.1%; figure 3*d*).

#### 3.2.3. Post-conflict context with a former opponent

Post-conflict context with an opponent substantially increased signalling probability and complexity during approaches in both chimpanzees and mangabeys (electronic supplementary material, tables S11–S14).

**Figure 4.**
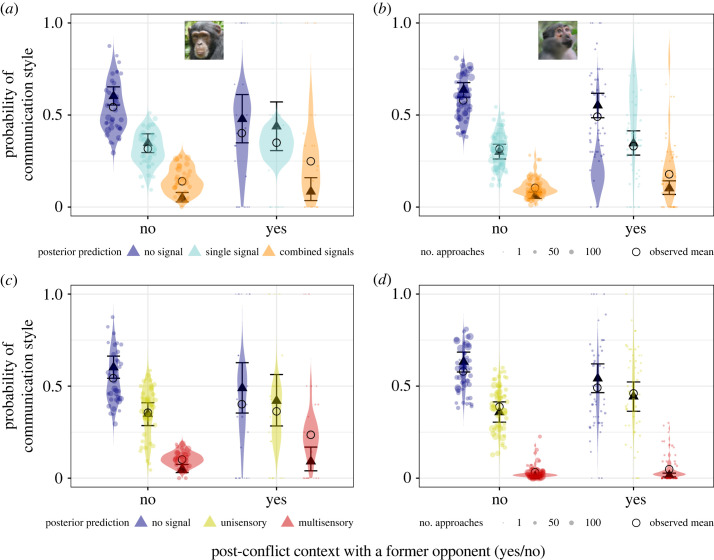
Post-conflict contexts with a former opponent increased signalling probability and complexity during social approaches in both chimpanzees and mangabeys. (*a* and *b*) Probability of emitting single and combined signals during approaches, (*c* and *d*) probability of emitting unisensory (visual or auditory) and multisensory signals during approaches: in chimpanzees (left) and mangabeys (right). Triangles: posterior means; error bars: 95% credible intervals; dots: individual probabilities from the raw data (size: number of approaches sampled per individual per context), violin: predicted individual probabilities from the posterior distribution of the model.

Chimpanzees were 60% more likely to use combined signals in post-conflict approaches with a former opponent compared with the rest of the approaches (combined versus no signal, 0.69, 95% CI [0, 1.38], *p +* = 97.3%; combined versus single signals: 0.32, 95% CI [−0.32, 0.95], *p +* = 83.7%), but we found no evidence of a consistent effect on the probability to use single signals (single versus no signal, 0.45, 95% CI [−0.1, 1], *p +* = 94.1%; figure 4*a*). Chimpanzees were 117% more likely to emit multisensory signals in post-conflict approaches with a former opponent compared with the rest of the approaches (multisensory versus no signal, 0.74, 95% CI [0.05, 1.42], *p +* = 98.4%; multisensory versus unisensory signal: 0.51, 95% CI [−0.16, 1.15], *p +* = 93.5%) but we found no evidence of a consistent effect on the probability to emit unisensory signals (unisensory versus no signal, 0.37, 95% CI [−0.19, 0.94], *p +* = .0%; figure 4*c*).

During post-conflict approaches with an opponent compared with the rest of the approaches, mangabeys were 17% more likely to emit single type signals (single versus no signal, 0.3, 95% CI [0.06, 0.54], *p +* = 98.9%), and 76% more likely to emit combined signals (combined versus no signal, 0.62, 95% CI [0.29, 0.94], *p +* = 100%; combined versus single signal: 0.33, 95% CI [0, 0.64], *p +* = 97.8%; figure 4*b*). In post-conflict approaches with an opponent compared with the rest of the approaches, mangabeys were also 26% more likely to emit unisensory signals (unisensory versus no signal, 0.38, 95% CI [0.14, 0.62], *p +* = 99.9%) and there was some support that mangabeys were 109% more likely to emit multisensory signals (89% CI not overlapping 0: multisensory versus no signal, 0.51, 95% CI [−0.03, 1.03], *p +* = 96.7%; multisensory versus unisensory signal: 0.12, 95% CI [−0.4, 0.62], *p +* = 69.2%; figure 4*c*).

#### 3.2.4. Third-party post-conflict context

Post-conflict interactions with a third party more markedly increased signalling complexity during approaches in chimpanzees than in mangabeys (electronic supplementary material, tables S11–S14).

**Figure 5.**
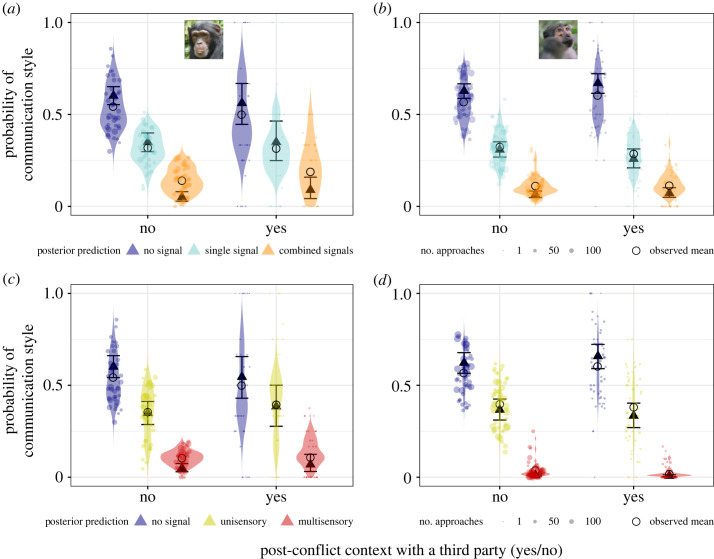
Third-party post-conflict contexts markedly increased signalling complexity during social approaches: combined signal production increased in chimpanzees but less so in mangabeys. (*a* and *b*) Probability of emitting single and combined signals during approaches, (*c* and *d*) probability of emitting unisensory (visual or auditory) and multisensory signals during approaches: in chimpanzees (left) and mangabeys (right). Triangles: posterior means; error bars: 95% credible intervals; dots: individual probabilities from the raw data (size: number of approaches sampled per individual per context), violin: predicted individual probabilities from the posterior distribution of the model.

Chimpanzees were 65% more likely to emit combined signals in post-conflict approaches with a third party compared with the rest of the approaches (combined versus no signal, 0.58, 95% CI [0.03, 1.11], *p* + = 97.9%; combined versus single signal: 0.54, 95% CI [0, 1.07], *p +* = 97.4%), but we found no consistent effect on the probability to emit single signals (single versus no signal, 0.03, 95% CI [−0.42, 0.48], *p +* = 55.6%; figure 5*a*). We found no evidence that post-conflict contexts increased the probability of emitting unisensory (unisensory versus no signal, 0.17, 95% CI [−0.25, 0.59], *p +* = 78.5%), nor multisensory signals in chimpanzees (multisensory versus no signal, 0.35, 95% CI [−0.27, 0.94], *p +* = 86.7%; multisensory versus unisensory signal: 0.21, [−0.38, 0.78], *p +* = 76.5; figure 5*c*).

During post-conflict events with a third party compared with the other approaches, mangabeys were 8% less likely to emit single type signals (single versus no signal, −0.24, 95% CI [−0.45, −0.03], *p* − = 99.3%) and there was some support that mangabeys were 15% more likely to use combined signals (combined versus no signal: 0.04, [−0.26, 0.33], *p +* = 57.8%; 89% CI not overlapping 0: combined versus single signal, 0.25, 95% CI [−0.05, 0.54], *p +* = 94.9%; figure 5*b*). In third-party post-conflict contexts compared with the other approaches, there was some support that mangabeys were 27% less likely to emit multisensory signals (89% CI not overlapping 0: multisensory versus no signal, −0.5, 95% CI [−1.12, 0.1], *p −* = 95.3%; multisensory versus unisensory signal: −0.36, 95% CI [−0.99, 0.23], *p* *−* = 88.1%). We found no clear evidence that post-conflict scenarios with a third party affected the probability to emit unisensory signals in mangabeys (unisensory versus no signal, −0.14, 95% CI [−0.34, 0.06], *p* *−* = 93.2%, figure 5*d*).

#### 3.2.5. Summary of the results

**Figure 6.**
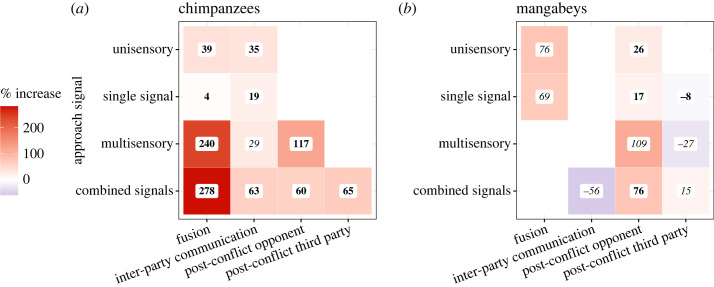
Summary of results: impact of socially uncertain contexts (fusion, inter-party communication, post-conflict contexts with a former opponent or with a third party) on signalling probability and complexity during social approaches. (*a*) Chimpanzees, (*b*) mangabeys: per cent change in probability of signalling style in socially uncertain contexts relative to the approaches occurring in other contexts, calculated based on estimates with credible intervals at 95%, bold: supported by 95% and italics by 89% of the posterior distribution).

Figure 6 summarizes the results of the models on socially uncertain contexts, and signalling probability and complexity during approaches (effects supported only by 89% of the posterior distribution are written in italic, those supported by 95% of the posterior distribution are written in bold). (*a*) In chimpanzees, contexts of fusion and post-conflict approach with a former opponent increased the probability of producing multisensory and combined signals. To a lesser extent, fusion contexts also increased the probability of producing unisensory and single signals in chimpanzees. (*b*) In mangabeys, there was some support that post-conflict contexts with a former opponent increased the probability of signalling during approaches, particularly related to combined and multisensory signals. By contrast, fusions increased the probability of producing unisensory and single signals in mangabeys. In chimpanzees, third-party post-conflict contexts increased the probability of emitting combined signals. In mangabeys, third-party post-conflict contexts decreased the probability of emitting single signals, and multisensory signals, while it increased the probability to emit combined signals. Inter-party communication events had a negative effect on the probability to emit combined signals in mangabeys, but increased signalling likelihood in chimpanzees, particularly regarding combined signals (for detailed results see the electronic supplementary material, tables S11–S14).

### 3.3. Impact of social relationship strength and dominance rank on signalling patterns

In both species, subordinate signallers were more likely to emit single or combined signals, and unisensory signals compared to dominant signallers. However, in mangabeys, dominant signallers were more likely to emit multisensory signals compared with subordinate signallers (electronic supplementary material, tables S11–S14 and figure S11). Overall, we found no evidence that social relationship strength (estimated by a GI) consistently modulated signalling variables during approaches in mangabeys and chimpanzees (electronic supplementary material, figure S12).

## 4. Discussion

In this study, we tested the hypothesis that social uncertainty, a component of social complexity, promotes signalling complexity in four groups of two sympatric primate species exhibiting different levels of fission–fusion dynamics, western chimpanzees, and sooty mangabeys [1,5,46]. The studied groups shared the same forest habitat, thereby limiting potential habitat-specific impacts on signalling strategies [34]. Comparing the two species, mangabeys showed a greater reliance on visual signals and chimpanzees a greater reliance on auditory and multisensory signals during social approaches. Here, the species facing higher predation pressure [99], mangabeys, more frequently used the visual rather than the more conspicuous auditory channel. In contexts of higher social uncertainty, both species showed a greater probability to signal and to use more complex signals, with the greatest impact of social uncertainty on signal complexity observed during chimpanzee fusion events. The higher fission–fusion dynamics, and thereby lower association predictability, in chimpanzees relative to mangabeys, may therefore underlie some of these general species' differences in signalling preferences.

### 4.1. Impact of socially uncertain contexts on signalling patterns

In both species, we found evidence that socially uncertain contexts increased signalling probability and complexity, thus supporting the hypothesised link between the complexity of the social environment and signalling patterns [1,5]. Considering that we examined signalling during social approaches that led to socio-positive or tolerant outcomes only, signals in this sample were unlikely to be threats or redirected aggressive signals. What advantages may be gained by using combined or multisensory signals during events that engender social uncertainty? High social uncertainty can trigger emotional and physical arousal and is related to an increased risk of conflict [29,30,36]. From the production perspective, additional signals may be beneficial in uncertain situations, as they can serve as a back-up strategy (signals having a redundant meaning or function) or reduce ambiguity by refining information conveyed (non-redundant signals), which allow them to limit message transmission or interpretation failures [33,34,66,70,73,100,101]. From the perception perspective, the combination of auditory and visual modalities may speed up detection and discrimination mechanisms by soliciting different perception channels in the receiver [32–34,62], thereby triggering more frequent, faster, or desirable responses [102–104]. Therefore, the observed increase in usage of both combined and multisensory signals during contexts associated with outcome unpredictability or high aggression risk (socially uncertain contexts) may both evolve to improve message efficacy, at the transmission, integration, or interpretation levels.

Animals flexibly employ communication as a social strategy to reduce social uncertainty. In post-conflict scenarios, for example, they do so in order to reconcile with a previous opponent during post-conflict approaches [39,42,105] or solicit intervention and consolation from third-party individuals [20,41]. Further, studies have shown that during fusions, wild chimpanzees combine vocal signals [85], and also combine vocal with visual signals when the risk of aggression is high, such as when greeting high-ranking conspecifics [30]. In both species, we found that post-conflict contexts with a former opponent increased the probability of signalling and emitting combined and multisensory signals. Similarly, third-party post-conflict events increased the probability of emitting combined signals in both species, but to a lesser extent than post-conflict contexts with a former opponent. In contrast with post-conflict contexts with an opponent, third-party post-conflict contexts decreased the use of multisensory signals in mangabeys. These findings in both species may indicate lower levels of social uncertainty in post-conflict contexts with a third party than with a former opponent. Finally, inter-party communication increased signalling probability and complexity in chimpanzees, but decreased signal combinations in mangabeys. Overall, these results suggest that third-parties in chimpanzees may play a more determinant role in stimulating combined signalling during dyadic approaches than in mangabeys. However, given the limited sample size of approaches following inter-party communication events in mangabeys, we cannot rule out that these contexts may impact other aspects of signalling during approaches or within-party social behaviours. Fusions, compared to non-fusion events, impacted signalling complexity in both species but in different ways. Specifically, fusions increased mangabey likelihood to signal and chimpanzee likelihood to elicit more combined (signal types) and multisensory (auditory and visual) signals during approaches.

### 4.2. Fission–fusion dynamics as a driver of signal complexity in chimpanzees

Fission–fusion dynamics offer social advantages, such as the possibility to modulate individual gregariousness [106] and minimize conflicts of interest or conflict escalation [107–109], while also allowing the accrual of the benefits of group-living [110,111]. High degrees of fission–fusion dynamics probably lead to certain signalling adaptations, for instance the maintenance of long-distance auditory contact between subgroups, which allows maintaining dyadic and group coordination [112–115]. However, frequent changes in potential partner and audience composition triggers unpredictability in the social environment and thus promotes behavioural flexibility [2,19]. This study highlights that social complexity associated with high fission–fusion dynamics probably requires expanded communication skills and high behavioural flexibility in chimpanzees, and may explain some of the signalling differences observed between species.

As audible signals are expected to reach a wider audience than visual signals [47], the frequent use of auditory and multisensory signals overall during close approaches in chimpanzees may indicate that chimpanzees are more likely to direct information to both visible and non-visible audiences than mangabeys, for instance offering status updates to third parties about current social risk or subgroup membership, and enabling group coordination and cohesion [20,47,49,70,112,115]. Also, chimpanzees compared to mangabeys used more, and more diverse, audible and multisensory signals (electronic supplementary material, figures S9 and S10). In chimpanzees, this included the use of objects which generate noise but mask identity (such as branches, leaves, the ground, or body parts). As vocal signals reveal identity to the audience [22,47], noisy object use may serve to attract attention without revealing identity to potential eavesdroppers [70].

Inter-party communication events yielded increased signalling probability and complexity in chimpanzees, but not in mangabeys. The imminent arrival of others can create social uncertainty by changing within-party social dynamics [21,22]. Approaches following inter-party exchanges may reflect behaviour to advertise social bonds [49] and limit shifts in allegiance. Alternatively, inter-party communication can predict future party composition and fusion, as calls can give information on individual identity, location and activity [51,52]. As such, signalling may mediate coordination mechanisms underpinning fission–fusion dynamics in this context [53]. Thus, while social uncertainty during fusions may encompass high unpredictability of the social environment, such as a need to re-assess relationships and/or to reduce the risk of conflict [19,30,48], social uncertainty during and after inter-party communication events may relate more to changes in social dynamics and movements and the risk of losing a social partner [53,112].

To conclude, although both species live in large groups, complex communication may be a more crucial social tool for the maintenance of social relationships and group cohesion in chimpanzees compared with mangabeys, owing to the higher degrees of fission–fusion dynamics [3,116,117]. High fission–fusion dynamics are apparent in several taxa, with varying degrees of differentiation in social relationships [45,46], and are a common feature of human societies [118]. As fusion, inter-party communication, and post-conflict mechanisms may generate social uncertainty but ultimately ensure group cohesion [42,48,112], future research could compare signalling complexity during these contexts across multiple species or groups exhibiting varying levels of fission–fusion dynamics, but also levels of relationship differentiation, as a way to evaluate the trade-off between relationship repair and dispersion when the risk of conflict is high [105,119].

### 4.3. Impact of social relationship strength and dominance rank on signalling patterns

At the dyadic-level, lower-ranking signallers in chimpanzees, and less markedly so in mangabeys, more frequently emitted combined signals compared to single signals, when interacting with a higher-ranking individual. This finding adds to our results supporting a positive effect of social uncertainty on signalling complexity, presumably used by low-ranking individuals to improve transmission efficacy or reduce message ambiguity during approaches. This is consistent with previous studies, where low-ranking individuals more frequently used signal combinations in chimpanzees [117], such as pant-grunts and pant-barks [30,54]. As we focused on approaches that led to affiliative or tolerant outcomes in this study, our findings suggest that complex greeting signals may allow conflict prevention or coordination [30,120]. We also found that dominant individuals in mangabeys, relative to subordinates, showed a higher tendency to emit multisensory signals during approaches with non-agonistic outcomes. That higher ranking mangabeys increased signal complexity while approaching subordinates provides some support for the ‘benign intent’ hypothesis [23,55].

We did not find strong support for the hypothesis that social interactions with unfamiliar individuals require more complex signals because of the higher social uncertainty between these partners [55]. Social aspects unrelated to uncertainty, like a need of cooperation maintenance, may also elicit complex signals. For example, wild male geladas are more likely to produce combined signals before grooming with female partners [63]. Further, complex signalling displays directed towards strong allies may also serve to reinforce and advertise social bonds after prolonged separation events, as suggested in wild black-horned male capuchins [49]. Thus, our findings in both species are consistent with previous studies that found contrasting results linking social relationship strength and signalling complexity [30,31,49,54,55,87].

### 4.4. Limitations of this study and future directions

In order to obtain a large dataset representative of social approaches across a wide range of contexts and dyads and extended time spans, we chose to focus on an observational approach. Using video and acoustic data would have given a more detailed assessment of signals, but it would have limited the number of approaches and contexts we could capture. To accommodate this, we chose a conservative signal coding scheme with well-defined and conspicuous signal categories (i.e. detectable irrespective of the position of the human observer), that can be reliably coded, as demonstrated by high inter-rater reliability scores. While this conservative approach may under-represent signal complexity, it was consistent across groups and species and therefore we are confident that it did not bias our results. Future research could examine signal use in more detail, for instance including sequence length, signal intensity or variants, distinguishing multisensory signals containing a single type or combined types, differentiating overlapping multisensory from sequential multisensory signal combinations, and further examining signal specificity across contexts [31,32,66,79]. Particularly, increases of single/unisensory signals were also detected during fusions and post-conflict approaches with an opponent compared with other approaches in both species, although showing a smaller magnitude of change than for combined signalling. Thus, emitting single signals may also offer a strategy to reduce message ambiguity [39]. The results of how dominance and social relationships shape signalling complexity in approaches between opponents after conflict may also vary depending on aggression intensity or the methodological approaches used to quantify relationship quality, which we did not examine here [42,105,121]. Future studies should consider the influence of kinship on signalling patterns, which we did not explore here because of a lack of kinship data for all individuals. Moreover, future studies could explore the influence of ontogeny on the use and development of signal combinations during these contexts, as young ages may increase social uncertainty [122], or alternatively be associated with decreased social complexity [79] and costs of signalling ambiguity [100].

## 5. Summary and conclusion

In both mangabeys and chimpanzees, we assessed how individuals change their signalling during daily life events of varying contexts of social uncertainty, specifically during approaches that lead to socio-positive or tolerant outcomes. Fusion and inter-party communication events increased signalling complexity during approaches in chimpanzees, but not in mangabeys. Fusion was the most important factor influencing signalling complexity in chimpanzees, generating increased production of both combined and multisensory signals in this context. By contrast, post-conflict contexts with a former opponent elicited increased likelihood of signalling, and signalling complexity in both western chimpanzees and sooty mangabeys. However, post-conflict events with a third party affected signalling patterns more clearly in chimpanzees than in mangabeys.

In summary, moments of high social uncertainty increased signalling complexity in both species [1,24,27,31], although more so in chimpanzees. Particularly, this study pinpoints the impact of strong fission–fusion dynamics on signalling probability and complexity in chimpanzees [45], with signal complexity demonstrated in both multimodal and multi-signal capacities. Both within and between-species findings support the hypothesis that social complexity requires signalling flexibility, particularly regarding signal combinations, and thus signalling complexity [1,4,5]. As such, it is possible that through hominid evolution, societies with high levels of fission–fusion dynamics became more ubiquitous, which may have contributed to an increase of social and communicative complexity levels [45,118].

## Data Availability

Data (doi:10.6084/m9.figshare.23725722) and Rcode (doi:10.6084/m9.figshare.23723961) are provided on Figshare. Supplementary tables and figures are provided in the electronic supplementary material [123].
